# The Modulation of the Cell-Cycle: A Sentinel to Alert the NK Cells of Dangers

**DOI:** 10.3389/fimmu.2013.00325

**Published:** 2013-10-07

**Authors:** Florence Baychelier, Vincent Vieillard

**Affiliations:** ^1^UMR-S 945, Laboratoire Immunité et Infections, INSERM, Paris, France; ^2^UMR-S 945, UPMC Université Paris 06, Paris, France

**Keywords:** NK cells, activating receptors, stress, cell-cycle, infections, tumors

## Abstract

Natural killer (NK) cells are an essential component of innate immunity that provides a rapid response to detect stressed, infected, or transformed target cells. This system is controlled by a balance of inhibitory and activating signals transmitted by a myriad of receptors and their specific ligands. Inhibitory receptors mainly recognize self-MHC class-I molecules, whereas activating receptors, such as natural cytotoxic receptors, NKG2D, and DNAM-1, interact with self-proteins, normally not expressed on the cell surface of healthy cells, but up-regulated by cellular stress or infections and are frequently expressed on tumor cells. In these circumstances, regulatory controls ensure that specific ligands are induced mainly in diseased cells and not in normal cells. Each ligand seems to exhibit some distinct specializations providing broad “coverage” for numerous stresses associated with various diseases. Deregulated cell proliferation is a hallmark of these abnormal situations, and may serve as a sentinel for the elimination of the targets by NK cells. The purpose of this review is to discuss recent implications of cell-cycle to create a warning control system that relays various danger signals via specific ligands to the NK receptor system.

## Introduction

**Figure 1:**
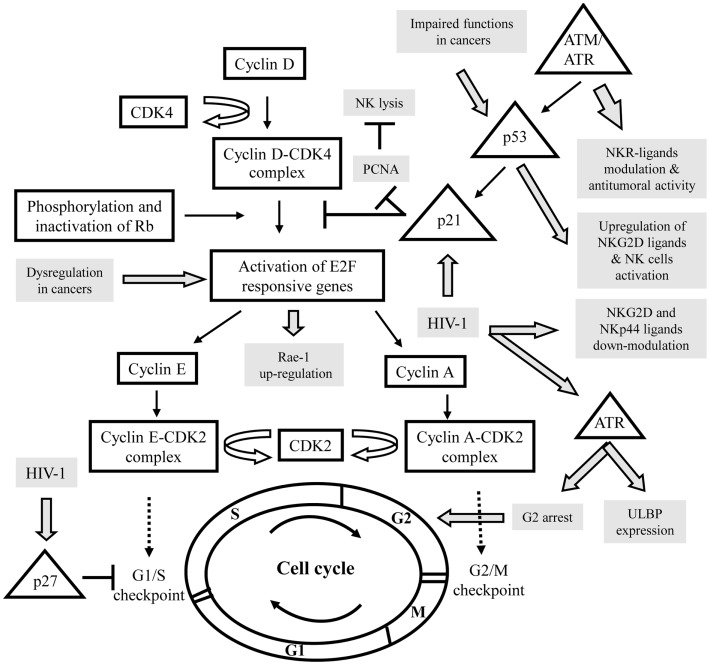
**Schematic representation of the interactions between the control of the cell cycle and the expression of specific ligands for activating NK receptors in various pathologies**. Cell-cycle actors are in squares and DNA damage pathway in triangles. The modulation of activating NK ligands expression in cancers or viral infections is indicated in gray. CDK, cyclin-dependent kinase; Rb, retinoblastoma tumor suppressor gene; ATM, ataxia-telangiectasia-mutated; ATR, ataxia, and rad3 related; PCNA, proliferating cell nuclear antigens.

Natural killer (NK) cells are key components of the innate immune system, known to display strong cytolytic activity against tumor- or virus-infected cells, as well as immunoregulatory functions that influence adaptive immune responses (1, 2). Their functions are regulated by a series of surface receptors transducing inhibitory or activating signals. Inhibitory receptors are mainly represented by killer immunoglobulin-like receptors (KIRs), CD94/NKG2A, and ILT-2; they all recognize self-molecules of the HLA class-I repertoire, constitutively expressed on host cells. To destroy a target, NK cells are also composed of several activating receptors, like NKG2D, DNAX accessory molecule-1 (DNAM-1), an adhesion molecule physically and functionally associated to LFA-1 (Lymphocyte function-associated antigen 1), and the natural cytotoxicity receptors (NCRs; NKp30, NKp44, and NKp46) (3–5). Simultaneous interactions of certain of these activating receptors on NK cells with their specific ligands on the target cells lead to the integration of different intracellular signals, which together both dictate the quality and intensity of the effector NK cell response. The relative contribution of each of the activating receptors to NK cytotoxicity against target cells differs, indicating the existence of specific ligands. Indeed, multiple stress inducible cellular proteins have been identified. The NKG2D ligands comprise the MHC class-I chain related antigens (MIC-A and MIC-B) and the UL16 binding proteins (ULBP-1-6) (5), whereas, DNAM-1 recognizes the poliovirus receptor (PVR) and Nectin-2 (6). The identity of ligands for NCRs is a field of intense investigations; nuclear leukocyte antigen-B-associated transcript 3 (BAT3) factor, which is released from tumor cells under stress conditions, and a member of the B7 family, B7-H6, only expressed on tumor cells, both have been identified as cellular ligands for NKp30 (7, 8). Concomitantly, we have recently identified a cellular ligand of NKp44, called NKp44L, which is a novel isoform of the mixed-lineage leukemia-5 (MLL5) protein, expressed on a large panel of the tumor and transformed cells, and induced during Human immunodeficiency virus 1 (HIV-1) infection (9, 10). These virus-, stress-, or transformation-inducible ligands are normally not expressed on the cell surface of cells from healthy individuals, although in certain conditions high level of ligands can be found in healthy cells, such PVR on vascular endothelial cells (11), or NKp44L on articular chondrocytes (12). This suggests however, that small changes in their cell-surface profile may significantly influence the susceptibility of these targets to the NK cells. This is consistent with the idea that their expression needs to be regulated at different levels, including epigenetic, transcriptional, and posttranscriptional mechanisms. These levels of regulation may serve as serial checkpoints to ensure that ligands are induced only in diseased cells and not in normal cells. To date, however, this phenomenon is only partially understood. Recent insights in this field suggest that cell-cycle progression could be used as an endogenous indicator for potential danger for limiting tumor growth and viral replication. The cell-cycle machinery is distinguished by a series of coordinated events essential to ensure faithful DNA replication and segregation of replicated chromosomes into two separate cells. Replication of DNA occurs during the S phase. The S phase is preceded by the G1 phase during which the cell is preparing for DNA synthesis. The active replication of the chromosomes occurs during the S phase. Subsequently, the cells enter a further G2 gap period, prior to chromosome segregation and cytokinesis in M phase (mitosis), followed by an immediate re-entry into G1 (Figure 1) (13, 14). Cells in G1 can, before commitment to DNA replication, enter a quiescent state, called G0, when they have reversibly withdrawn from the cell-division cycle in response to high cell density, mitogen deprivation or some other stress situations. Alternatively, the cells may irreversibly withdraw from the cell-cycle into terminally differentiated or senescent states. Progression through each cell-cycle phase and transition from one phase to the next are monitored by specific checkpoints, which maintain the correct order of events (15). If these sensors detect aberrant or incomplete cell-cycle events (e.g., DNA damage), checkpoint pathways carry a signal that can trigger cell-cycle arrest until the problem is solved (16, 17). These checkpoints are governed by a tight relationship of specific cyclin-dependent kinase (CDK), and their associated inhibitor partners of the Cip/Kip family proteins (p21, p27, p57), which can reversibly halt cell-cycle progression (Figure 1) (18). In addition, different protein kinases are specific to DNA damage, like ataxia-telangiectasia-mutated (ATM), ataxia, and rad3 related (ATR). These kinases phosphorylate p53 in response to DNA damage, resulting in p21 blocking the cell-cycle, at least at the G1/S checkpoint (19). Failure of the checkpoints to arrest the cell-cycle may result in genetic instability, contributing to uncontrolled proliferation implicated in cancer development and viral replication. Mitogens release the brakes of cell-cycle progression by stimulating G1/S CDK activities, which trigger the phosphorylation of retinoblastoma tumor suppressor gene product (pRb) proteins, leading to disruption of their interaction with the E2F family of transcription factors, which play a crucial role in the control of the cell-cycle (Figure 1) (20).

It was shown that NK cells have the ability to bind target cells in mitosis and to attack pathogenic cells in mitosis more effectively than the same cells in other stages of the cell-cycle. Thus, cells in mitosis warrant and undergo heightened surveillance, a novel strategy for immunologic assessment of danger (21). For example, the cellular ligands of NKp30 (NKp30L) and NKp44 (NKp44L) were cell-cycle regulated; in the presence of colchicine or nocodazole their expression is specifically reduced in target cells arrest in G2/M phase, suggesting their downregulation during mitosis (22). Similarly, stress signals cause surface expression of NKG2D ligands (NKG2D-L) via sensors of DNA damage, like ATM/ATR kinases, triggering signaling cascade, which leads to cell-cycle arrest (Figure 1). (5).

## Relevance of Cell-Cycle Dysfunctions to Trigger NK Lysis

### In cancers

A tight connection between the cell-cycle and cancer is obvious, because cell-cycle machinery controls cell proliferation, and in stark contrast to normal cells, which only divide a delimited number of times before they enter in growth arrest, whereas, cancer cells never cease to proliferate. It appears that tumor suppressor gene mutations are highly likely to promote, and may even be required for, a large number of spontaneous and hereditary forms of cancer. Loss of function of the tumor suppressor gene product pRb, for example, would be predicted to liberate E2F transcriptional activators without requiring phosphorylation and thus bypass a normal negative regulation controlling entry into the cycle. E2F is expressed in response to growth factor stimulation and oncogenic stress and induce transcription of target genes involved in cell-cycle progression and DNA replication. As a result of its critical role in proliferation, the regulatory pathway for E2F is one of the most deregulated pathways in cancer (23). Recently, Raulet’s group have demonstrated in mice that expression of retinoic acid early inducible gene 1 (Rae-1), an NKG2D-L, in cancer cell lines and proliferating normal cells is coupled to the cell-cycle regulation Thus, Rae-1 is directly transcriptionally activated by E2F family transcription factors, which control the G1/S transition, and then play a central role in the cell-cycle entry (24). Liu et al. (25) have shown that upregulation of Rae-1 in mouse and ULBP-1 in human are dependent of the Ras pathway; an over-expression of the constitutively active HRasV12 mutant, which accelerates cell-cycle progression, seems sufficient to induce NKG2D-L expression and then to increase sensitivity of cells to NK-cell-mediated cytotoxicity (Figure 1). It is however important to note the conflicting roles of NKG2D and its ligands in cancer, which can promote the destruction of malignant cells or alternatively can be exploited for immune evasion (26).

In addition to regulating cell-cycle progression, E2F exerts a role in the DNA damage response by inducing target genes that function in DNA repair and recombination and DNA damage checkpoints. E2F is directly phosphorylated by ATM and ATR protein kinases that led to elevated mRNA expression of certain NKG2D-L (27–29). Knockdown studies established that constitutive ATM or ATR activation has an important role in maintaining constitutive ligand expression, and then to mobilize NK cells against the tumor (30). These studies indicated that NKG2D-L expression on tumor cells is mediated in part by an activated DNA damage response. On another hand, it is well established that the ATM/ATR pathway activates p53 (Figure 1). The tumor suppressor protein p53 is a sequence-specific DNA-binding protein that is able to induce either arrest or apoptosis at the cell-cycle checkpoints. Consistent with a prominent role as a tumor suppressor, p53 is mutated or deleted in approximately 50% of cancers, and in the remaining tumors, p53 function is frequently impaired (31). The deletion of p53 in various tumor cell lines indicated that p53 is certainly not essential for ligand expression, although p53 can induce certain ULPBs (5, 32). By using ChIP and luciferase reporter assays, they show that ULBP-1 and -2 are direct p53 target genes. Indeed, targeting of p53 by small molecular compounds, like RITA and Nutlin-3, up-regulated ULBP2 expression in sarcoma but not in carcinoma cell lines (33, 34). This could be explained by cell type-specific DNA methylation or histone deacetylation of the ULBP-1 or -2 promoters, and then their accessibility to transcription factors, such as p53. In line with these data, Fiegler et al. (35) have recently shown that B7-H6 expression is downregulated in various tumor cell lines upon treatment with histone deacetylase (HDAC) inhibitors or after siRNA-mediated knockdown of HDAC-2 or -3. Notably, the downregulation of B7-H6 on tumor cells by HDACi reduces NKp30-dependent effector functions of NK cells. Thus, in this context, it would be interesting to therapeutically evaluate different drug combinations, which induce cell-cycle arrest, including DNA damage-inducing drugs and HDAC activators to modulate ligand’s expression of activating NK receptors.

### In viral infections

Many cellular pathways are activated early on upon viral infection to achieve a state of survival and increased cellular proliferation for optimal replication and production of progeny virus. The induction of cell-cycle checkpoints and activation of the ATM/ATR dependent pathway have been reported to accompany infection by a number of different viruses (36).

Human immunodeficiency virus 1 is a prototypic example to study the implication of the cell-cycle in the control of the NK pathway. Indeed, several groups have demonstrated that during infection, ligands of activating receptors are significantly modulated, consistently to the NK lysis susceptibility (9, 37). In parallel, HIV-1 has developed numerous strategies to evade the activation of NK cells and have influenced the evolution of NK cell receptors and their ligands (38). During HIV-1 infection, DNA damage sensor ATR but not ATM is phosphorylated and activated by the viral protein Vpr (39). One of the benefits of ATR’s induction for HIV-1 is to induce G2 arrest (Figure 1) (40). This is associated with Vpr binding to damaged DNA-binding protein I (DCAF1), a substrate recognition subunit for an E3 ubiquitin ligase complex (41). ATR’s phosphorylation through Vpr not only results in G2 arrest, but also leads to the expression of ULBP-1 and -2, but not ULBP-3-6, or MIC-A and -B (42). The specificity of NKG2D-L expression may be due to ATR’s influence on the specificity protein (SP)1 and SP3 transcription factors resulting in ULBP-1 or -2 transcription (43).

Concomitantly, to facilitate viral persistence, Nef protein delays the progression of CD4^+^ T cells through the G1/S phase checkpoint control of the cell-cycle, involving an upregulation of p21 and p27 CDK inhibitors and the downregulation of cyclin D1 and cyclin A that promotes immune evasion and cell survival (44). Nef is also implicated in the down modulation of NKG2D-L (28), and NKp44L (45). Thus, the regulation of ligands for activating NK receptors expression by Vpr, Nef, and possibly other viral proteins might have different impacts on NK cell recognition of HIV-infected CD4^+^ T cells. To date, direct implication of the cell-cycle in this phenomenon remains unknown, however, Madrid and Ganem (46) recently showed that during lytic Kaposi’s sarcoma-associated herpesvirus (KSHV) infection, the subcellular localization of NKp44L changes; its expression on the cell surface decreases, as we and others have previously shown in HIV-1-infected CD4^+^ T cells (45). More intriguingly, they showed the total cellular NKp44L level did not change: it was instead mislocalized, concentrated in the nucleus. Intriguingly, KSHV is known to encode a viral cycle, denoted K-cyclin, extraordinary by its ability to mimic cellular components of the cell-cycle and play with him. Understanding the role of the virally encoded cyclin on the ligand’s expression represents an exciting problem for the future.

### In cell-to-cell cross-talks

In addition to their role in providing antitumor and antiviral immunity, NK cells are also able to regulate the adaptive immune response by secreting different cytokines and chemokines (2). Several studies have also provided evidence of cognate cell–cell interactions between NK and other immune cells. Although, expression of ligands for activating NK receptors was thought to be mostly restricted to transformed-, infected-, and/or stressed-cells, experimental evidences report that certain ligands can be also expressed in normal situations, on bone-marrow cells, dendritic cells, and B or T lymphocytes, possibly to regulate certain cellular responses (28, 47, 48). The implication of activating receptors expressed by NK cells in the direct elimination of T cells remains still elusive, although they have been recently focus of intense research (49).

DNAM-1-L and NKG2D-L were mainly expressed on T cells that had gone through at least one mitosis (28, 50). However, the expression of NKG2D-L on activated CD4^+^ and CD8^+^ T cells, in response to super-antigens, alloantigens or to a specific antigenic peptides, is variable, depending on the donors, but also on the time-stimulation of T cells and the experimental settings (28). Other and us have also reported this variability of cell-surface expression, for ligands of NCRs and NKG2D in various pathologic situations (22, 45). The expression and function of PVR, a ligand of DNAM-1, another activating NK receptor, was also investigated on T cells in response to super-antigen stimulations (50). Its expression is almost exclusively confined to cells that are in the S or G2/M phases of the cell-cycle and that have undergone at least one division as measured by the loss of CFSE intensity, a powerful tool to monitor lymphocyte proliferation and to quantify cell-division. In fact, the induction of PVR partially depends on ATM/ATR kinases pathway on activated T lymphocytes in a ROS-dependent manner. This could participate in the NK-cell-mediated recognition and lysis of proliferating T, by a specific PVR:DNAM-1 cross-talk. Conversely, oxidative stress has been previously found to induce several NKG2D-L, but not PVR (51). These findings highlight the complexity of the molecular mechanisms leading to the induction of different ligands for distinct NK-activating receptors, and further support the notion that they may involve common or distinct pathways, depending on the form of stress, the type of ligand, and the cellular context.

Thus, we could envisage that the expression of ligands for activating receptors on proliferating T lymphocytes is a possible mechanism used by NK cells to restrict the expansion of activated/proliferating T cells. In line with these data, the Swanborg’s group has recently shown that NK cells induce a reversible p21-mediated G0/G1 cell-cycle arrest of activated T cells, both in a contact-dependent, and an antigen non-specific manner (52). p21 has been shown to prevent cell-cycle progression by several mechanisms, including blocking formation of active cyclin-CDK complexes, and inhibiting DNA replication through proliferating cell nuclear Antigens (PCNA), which is commonly overexpressed in cancer cells, and contributes to cellular proliferation and transformation (Figure 1). Interestingly, PCNA was recently described as an unexpected inhibitory ligand for the NCR NKp44; an over-expression of PCNA was found to inhibit NK cell cytotoxicity in an NKp44-dependent manner, revealing a putative novel mechanism for pathologic cells to evade NK attack (53, 54), closed to the numerous mechanisms developed by tumor and infected cells to counteract their sensitivity to NK cells (55).

## Conclusion

As shown during this last decade, ligands for activating NK receptors exhibit distinct specializations in function of different stress situations, providing a complex warning system that relays various danger signals through the NK system. Uncovering the molecular mechanism that drives expression of each of these ligands remains an active area of research in the field. The potential linkage of these ligands to the cell-cycle control provides a new framework for future investigations designed to elucidate how their expression participates both in the immune homeostasis and disease control. However, current data suggest that for the cell-surface expressions of ligands; cell-cycle entry might be necessary but certainly not sufficient in most cases. Thus, it is tempting to speculate that compounds targeting the cell-cycle could also modulate the level of ligand’s expression. This could serve for the design of new agents to specifically control malignant cell proliferation or infections.

## Conflict of Interest Statement

The authors declare that the research was conducted in the absence of any commercial or financial relationships that could be construed as a potential conflict of interest.
